# Concurrent analysis of white matter bundles and grey matter networks in the chimpanzee

**DOI:** 10.1007/s00429-018-1817-8

**Published:** 2018-12-19

**Authors:** Rogier B. Mars, Jonathan O’Muircheartaigh, Davide Folloni, Longchuan Li, Matthew F. Glasser, Saad Jbabdi, Katherine L. Bryant

**Affiliations:** 1 Wellcome Centre for Integrative Neuroimaging, Centre for Functional MRI of the Brain (FMRIB), Nuffield Department of Clinical Neurosciences, John Radcliffe Hospital, University of Oxford, Oxford, OX3 9DU UK; 2 Donders Institute for Brain, Cognition and Behaviour, Radboud University Nijmegen, Nijmegen, The Netherlands; 3 Department of Forensic and Neurodevelopmental Sciences, Sackler Institute for Translational Neurodevelopment, London, UK; 4 Department of Neuroimaging, Institute of Psychiatry, Psychology, and Neuroscience, Sackler Institute for Translational Neurodevelopment, London, UK; 5 MRC Centre for Neurodevelopmental Disorders, King’s College London, London, UK; 6 Division of Imaging Sciences and Biomedical Engineering, Centre for the Developing Brain, St Thomas’ Hospital, King’s College London, London, UK; 7 Wellcome Centre for Integrative Neuroimaging, Department of Experimental Psychology, University of Oxford, Oxford, UK; 8 Marcus Autism Center, Children’s Healthcare of Atlanta, Emory University, Atlanta, GA USA; 9 Departments of Radiology and Neuroscience, Washington University Medical School, Saint Louis, MO USA

**Keywords:** Tractography, Connectivity, Great ape, Comparative, Brain organization, Networks, Temporal cortex, Frontal cortex, Diffusion MRI, Limbic system

## Abstract

Understanding the phylogeny of the human brain requires an appreciation of brain organization of our closest animal relatives. Neuroimaging tools such as magnetic resonance imaging (MRI) allow us to study whole-brain organization in species which can otherwise not be studied. Here, we used diffusion MRI to reconstruct the connections of the cortical hemispheres of the chimpanzee. This allowed us to perform an exploratory analysis of the grey matter structures of the chimpanzee cerebral cortex and their underlying white matter connectivity profiles. We identified a number of networks that strongly resemble those found in other primates, including the corticospinal system, limbic connections through the cingulum bundle and fornix, and occipital–temporal and temporal–frontal systems. Notably, chimpanzee temporal cortex showed a strong resemblance to that of the human brain, providing some insight into the specialization of the two species’ shared lineage.

## Introduction

Understanding the human brain in the context of evolution requires an appreciation of its similarities and differences to the brains of related species. Most comparative work, however, focuses on comparisons with just a few model species such as the macaque monkey or the marmoset. This is understandable, given that comparative studies on areal organization using, for instance, cytoarchitectonic or receptor-architectonic mapping, or on connections using tracers are time consuming, expensive, and often require data from a large number of individuals. For these reasons, as well as ethical concerns, data from the group of animals most closely related to humans, the great apes, is scarce. This seriously hinders our understanding of primate neural phylogeny.

One way to address this issue is to employ neuroimaging techniques in the study of comparative anatomy. Although neuroimaging does not have the resolution of some gold standard anatomical approaches, it has the advantages of providing whole-brain coverage in a reasonably short time without destroying tissue (Mars et al. 2014; Rilling 2014). Connectivity in particular is one aspect of brain organization that has been successfully studied using neuroimaging, using either diffusion MRI tractography or resting state functional MRI (Jbabdi et al. 2015). Connectivity data from neuroimaging have been used successfully to compare brain organization across species (Li et al. 2013; Mars et al. 2016b). One recent approach, for instance, uses an understanding of white matter architecture to provide a whole-brain comparison of brain organization between species (Mars et al. 2018c). This has the potential to formally compare the organization of the human brain to that of great apes such as the chimpanzee, identifying specializations in the different lineages.

Such an approach, however, requires a greater understanding of chimpanzee brain organization than is currently available. Although detailed studies have been performed on the arcuate fascicle (Rilling et al. 2008), the three branches of the superior longitudinal fascicle (Hecht et al. 2015), and the temporal connections of extrastriate cortex (Bryant 2015), a full understanding of chimpanzee cortical anatomy remains elusive with no modern atlases of the whole neocortex available. To address this problem, we here provide an explorative analysis of cortical grey matter networks and their associated white matter connections in the chimpanzee.

We benefited from two recent developments that now make such a study possible. First, we benefited from the public availability of high quality chimpanzee structural and diffusion MRI data via the National Chimpanzee Brain Resource. Second, we benefited from developments in exploratory data analysis of tractography data using independent components analysis that allow one to characterize both the grey matter networks of the cortex and their underlying white matter connections using a soft parcellation approach (O’Muircheartaigh and Jbabdi 2018). This enabled us to provide a first analysis of the organization of the entire chimpanzee cerebral cortex.

## Materials and methods

### Data

Data were made available by the National Chimpanzee Brain Resource (http://www.chimpanzeebrain.org) supported by the NIH National Institute of Neurological Disorders and Stroke. Data consisted of T1- and T2-weighted and diffusion-weighted magnetic resonance imaging data from 26 female chimpanzees (*Pan troglodytes*) acquired at the Yerkes National Primate Research Center (YNPC) on a 3T MRI scanner under propofol anaesthesia (10 mg/kg/h) using previously described procedures (Chen et al. 2013). All procedures were carried out in accordance with protocols approved by YNPRC and the Emory University Institutional Animal Care and Use Committee (Approval no. YER-2001206).

Two diffusion-weighted images (TR = 5900 ms; TE = 86 ms; 41 slices; 1.8 mm isotropic resolution) were acquired using a single-shot spin-echo echo planar sequence for each of 60 diffusion directions (*b* = 1000s/mm^2^), each with one of the possible left–right phase-encoding directions and 4 repeats, allowing for correction of susceptibility-related distortion. For each repeat of diffusion-weighted images, five images without diffusion weighting (*b* = 0 s/mm^2^) were also acquired with matching imaging parameters. These data have been used in previous reports (Bryant 2015; Chen et al. 2013). T1- and T2-weighted images were acquired on the same scanner with a 0.8 mm isotropic resolution.

### Preprocessing

T1- and T2-weighted images were processed using a modified version of the HCP pipeline (Donahue et al. 2016; Glasser et al. 2013). The HCP pipeline reconstructs the pial and white/grey matter interface surface by combining T1- and T2-weighted scans. Sample-specific surface templates (ChimpYerkes29 “Chimplate”) were first iteratively derived based on 29 chimpanzee data sets. The templates were then used as targets for registering the chimpanzee data sets employing a surface-based method.

Interhemispheric alignment between the left and right hemispheres in the templates was achieved using landmark-based alignment analogous to that performed on the macaque F99 atlas surfaces (Van Essen et al. 2012) using geographically corresponding landmark contours in each hemisphere. This procedure was justified by the observation that although hemispheric asymmetry exists in many areas of the brain, there is little ambiguity regarding what constitutes corresponding geographic features in the two hemispheres.

Diffusion MR data were analysed using tools from FSL (Smith et al. 2004; http://www.fmrib.ox.ac.uk/fsl). Images were skull-stripped using BET, with some manual correction, especially in the posterior occipital lobe. Diffusion-weighted MR data were corrected for eddy current and susceptibility distortion using FSL’s *eddy_correct* and *topup*.

### Tractography

Each dataset was prepared for probabilistic tractography using the bedpostX algorithm, modelling for up to three fibre populations per voxel. FSL’s probtrackx2 was used for probabilistic tractography (Behrens et al. 2007; Hernández et al. 2013), which was performed separately for each hemisphere by seeding from each of 20,252 cortical vertices of the mid grey matter surface, with 10,000 streamline samples initiated from each vertex, using the skull-stripped Chimplate brain template as target. To save on computation, the Chimplate target was resampled in 1.5 mm isotropic resolution. A stop mask was specified on the ipsilateral cortical pial surface to avoid the possibility of erroneous cross-sulcal fibres. The resulting dataset for each subject consisted of a connectivity matrix of streamline visitation counts for each of 40,504 cortical seed vertices to 171,557 possible brain target voxels. All subjects’ tractography matrices (referred to in FSL as “matrix2”) were subsequently averaged to create a group tractography matrix.

### Independent component analysis of tractography data

**Fig. 1:**
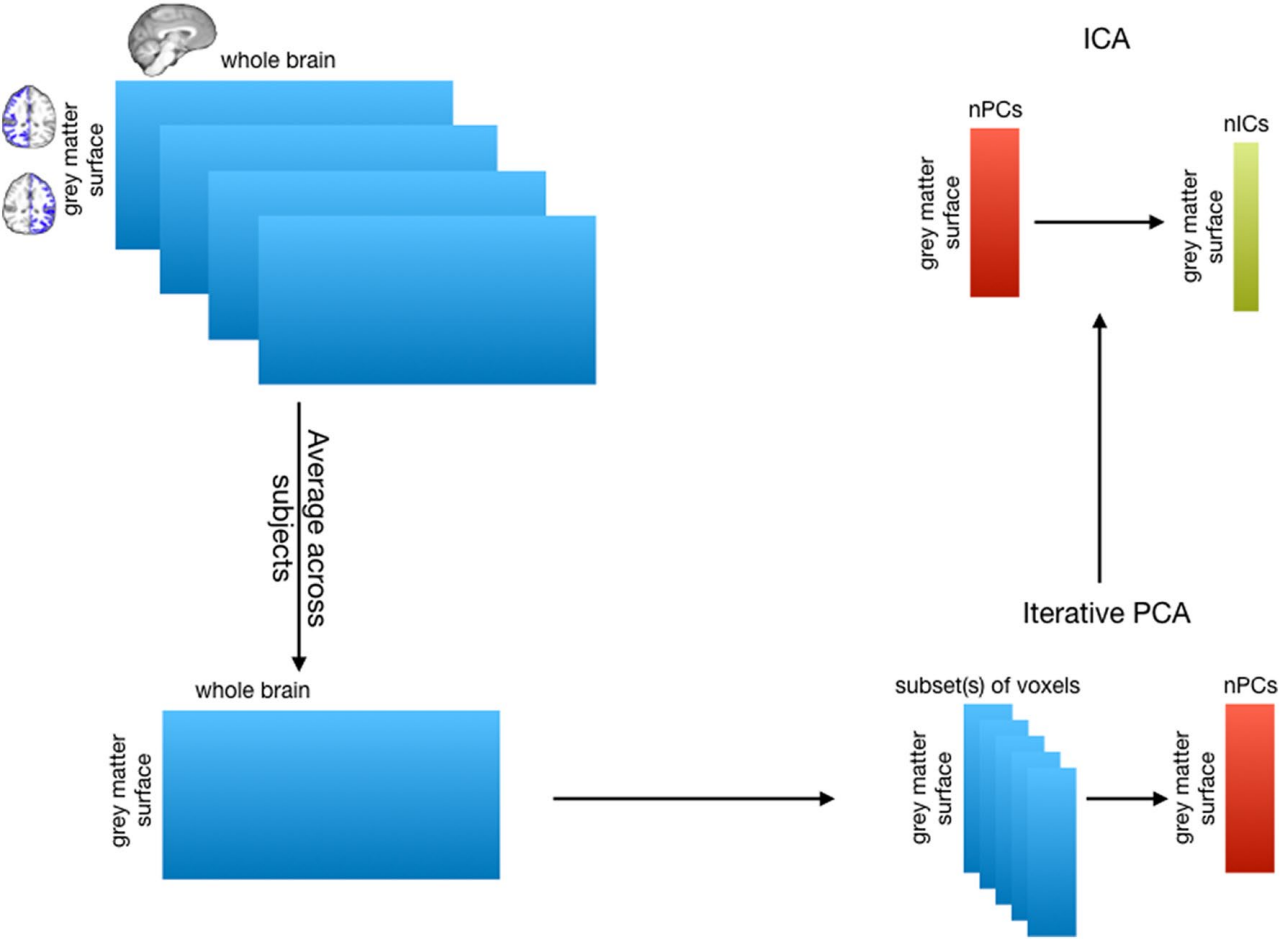
Independent component analysis on tractography matrices. (top left) Tractography was performed from the cortical surfaces to the whole brain (downsampled to 1.5 mm resolution). (bottom left) The resulting matrices were subsequently averaged to create one matrix of dimentions (grey matter vertices) × (whole brain voxels). (bottom left) This dimensionality of this matrix was iteratively reduced using PCA on a subset of the matrix. (top left) ICA was then performed on the reduced matrix

We sought to group together those vertices that shared a similar connectivity profile with the rest of the brain and identify their white matter connections. Following the logic that if two cortical surface vertices share a similar whole-brain connectivity profile, the rank of the group tractography matrix should drop by one, we can employ both principal component analysis (PCA) and independent component analysis (ICA) to group together vertices with similar whole brain connectivity profiles. Following the procedure developed by O’Muircheartaigh and Jbabdi (2018), we first reduce the dimensionality of the data using PCA and then use ICA to relax the orthogonality constrains of the PCA, allowing for instance spatial overlap between the components (Fig. 1). All these analyses were performed in Matlab (the Mathworks).

The PCA was performed using an adapted incremental approach (MIGP, proposed by Smith et al. (2014)) on the group tractography matrix. For each iteration of PCA, a matrix of all cortical seeds against a random subset of 10,000 whole brain target voxels was reduced to 4000 principal components, then a different random subset of 10,000 whole brain voxel values was concatenated to these 4000 eigenvectors (weighted by their corresponding eigenvalues) and PCA was run on this combined matrix, and so on until the whole brain has been covered. This way, only a relatively small matrix is analysed using PCA, making the procedure computationally realistic in normal computing environments.

ICA was performed on the resulting matrix using the fastICA algorithm (Hyvarinen (1999), http://research.ics.aalto.fi/ica/fastica/). We performed ICA using a fixed dimensionality (*K* = 50, following O’Muircheartaigh and Jbabdi 2018) with independence enforced in the seed domain, thus grey matter components were statistically independent from each other. This resulted in a set of K spatially independent maps in grey matter cortical regions with K associated spatial tractography profiles. Components were flipped as required to ensure the longest tail was positive. As these patterns were represented in the PCA subspace only, the normalized weighted ICs were projected back onto the full average tractography connectivity matrix using linear regression to reconstruct the whole brain tractography connectivity pattern. In addition, both the cortical surface and the volume were parcellated according to which component had the highest weighting in each vertex or voxel (i.e. winner-take-all), providing a hard parcellation of the cortical surface and whole brain volume. To assess the reliability of our results, the components in seed space and their white matter counterparts were fitted to a Gaussian/gamma mixture model as described in Beckmann (2012) and the positive gamma distribution was thresholded at *p* > 0.5.

Where possible we will refer to cortical territories using the nomenclature of Bailey et al. (1950) and sulcal nomenclature as used in Falk et al. (2018).

### Data availability

Analysis code and results images compatible with Connectome Workbench (Marcus et al. 2011) will be placed online in locations linked from the lab’s website (http://www.neuroecologylab.org) upon publication of the paper. Code is also included in the MR Comparative Anatomy Toolbox (Mr Cat). Raw data are available from the National Chimpanzee Brain Resource (http://www.chimpanzeeebrain.org).

## Results

**Fig. 2:**
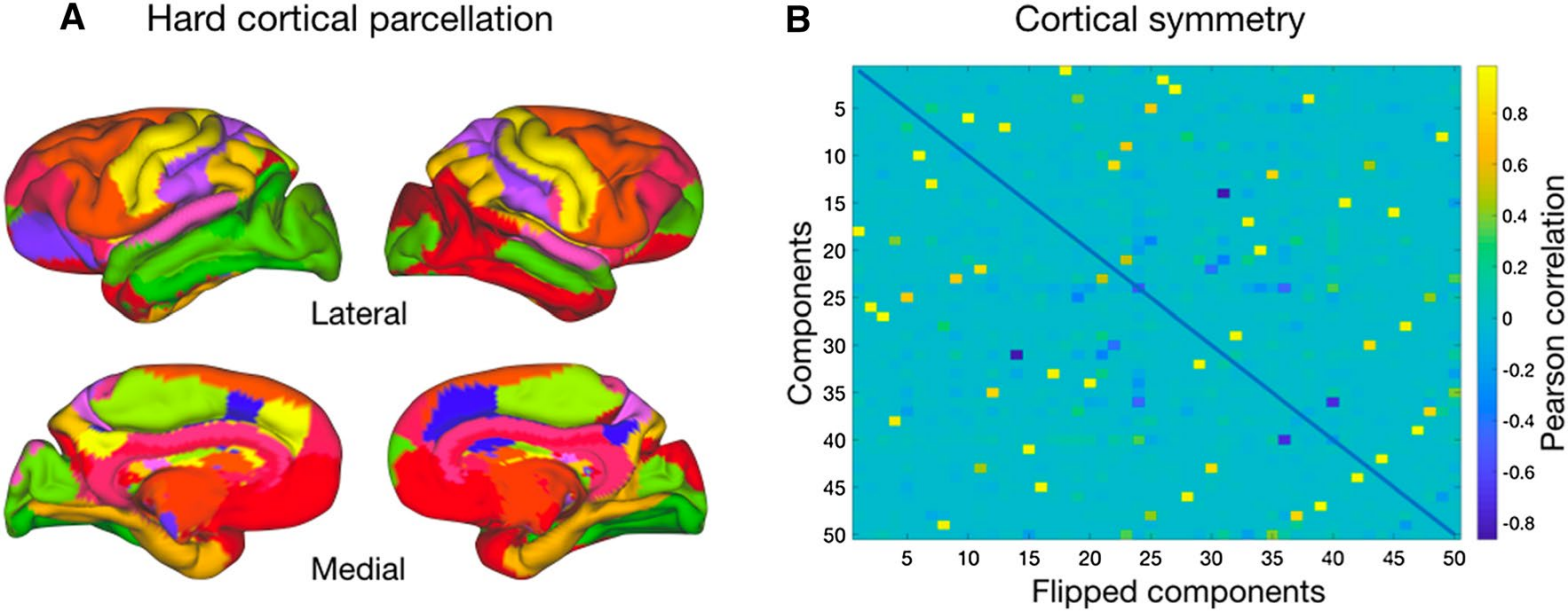
Cortical hard parcellation. **a** Cortical hard parcellation assigning each vertex the value of the component showing the strongest loading. For illustration purposes, strongly symmetric components were colored similarly in the two hemispheres. **b** Symmetry of components as assessed using spatial correlation of each component’s loadings on each vertex with the same measure for each component flipped across hemispheres

To make the results interpretable, we take the hard parcellation of the cortical surface as a starting point (Fig. 2a). To ensure reliability of the results we assessed the symmetry of each component by calculating its spatial correlation with all left–right flipped components (Fig. 2b) and we will concentrate our discussion primarily on symmetric components that capture similar organizational features in the two hemispheres. It should be noted that the symmetry, as calculated by the average of the highest spatial correlations of each component, was highest for the central sulcus components (*r* = 0.9372), intermediate for the limbic (*r* = 0.8277) and temporal–occipital (*r* = 0.8439) components, and noticeably lower for the dorsal components (*r* = 0.7279).

### Central sulcus

**Fig. 3:**
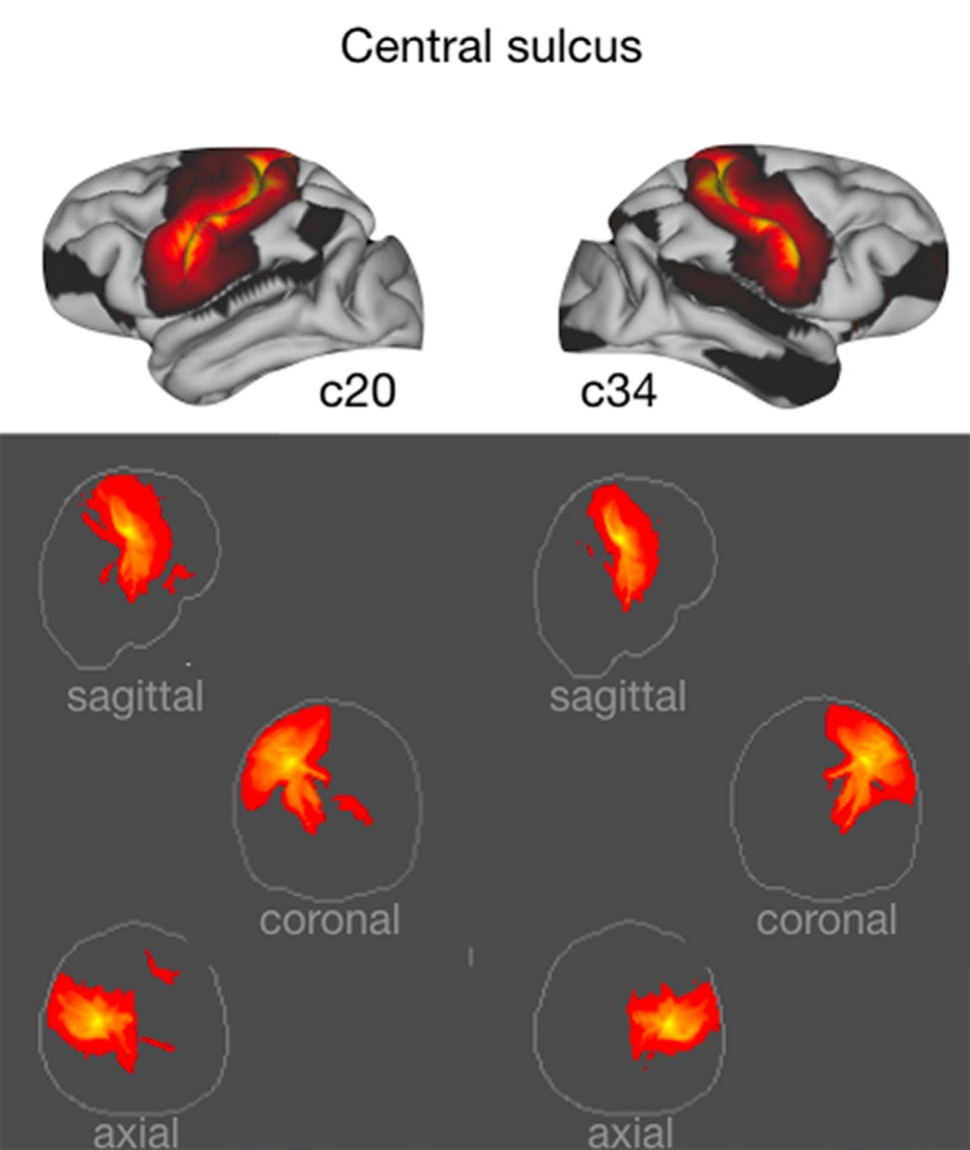
Central sulcus components. (top) Thresholded grey matter maps of components 20 and 34 on which vertices around the central sulcus maximally load. (bottom) Maximum intensity projections of the white matter connectivity of the two components, unthresholded for display purposes. All figures conventions are the same for all subsequent figures

A component with highest loading preferentially in the area around the central sulcus, and encompassing Bailey’s area FA in the frontal lobe, was evident in both hemispheres (c20 for the left hemisphere and c34 for the right hemisphere). These components showed highest spatial correlation with one another. The volume connectivity pattern of the components suggests that they were driven by the corticospinal tract (Fig. 3).

### Limbic system

**Fig. 4:**
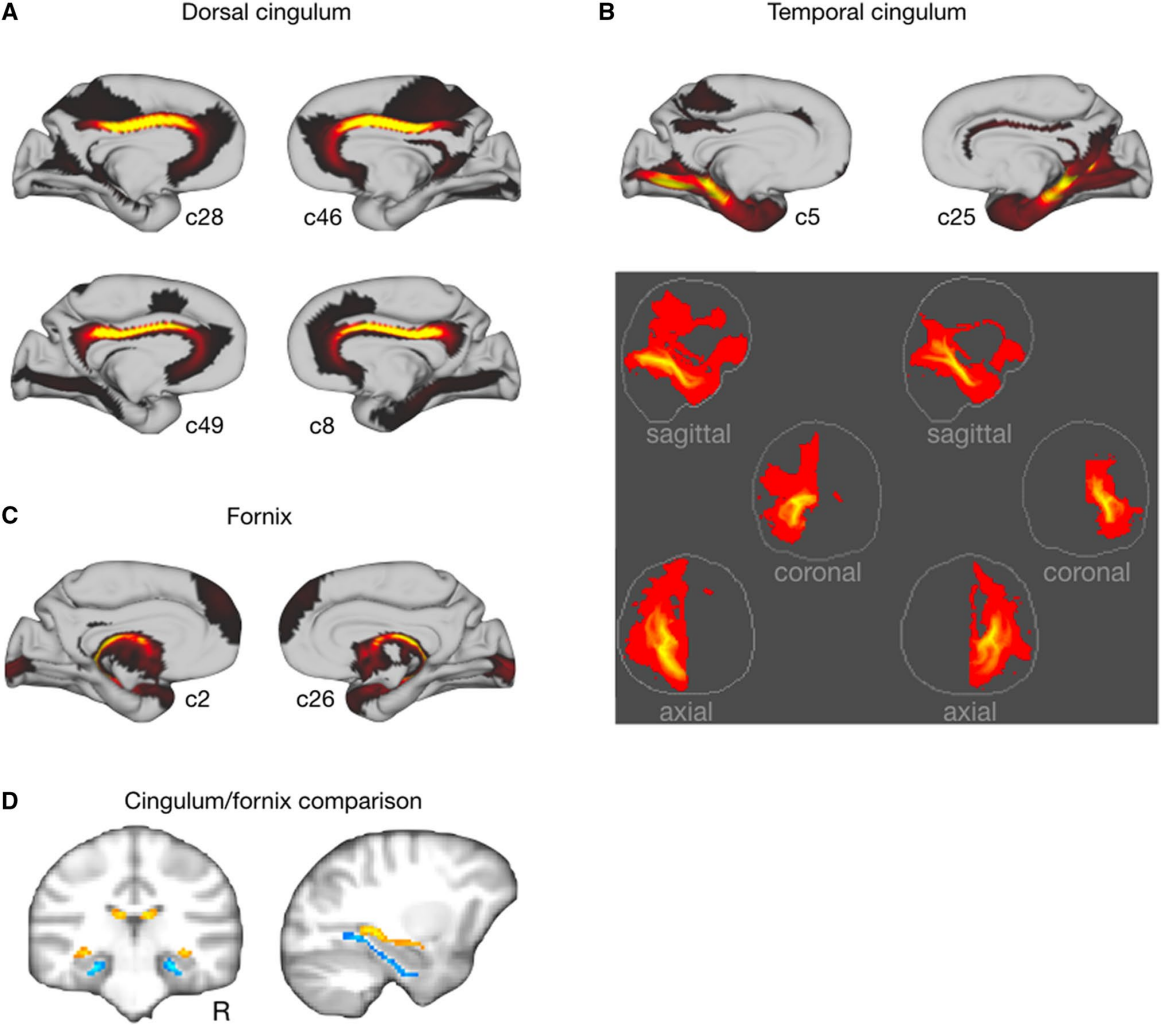
Limbic components. Grey matter maps and connectivity patterns of the components loading highest on **a** the dorsal territory of the cingulum bundle, **b** the ventral territory of the cingulum bundle, and **c** the fornix. **d** Thresholded connectivity patterns of the cingulum (yellow) and fornix (blue) components, illustrating their distinct course along the temporal lobe

A number of components showed a connectivity pattern similar to one or more of the limbic tracts (Catani et al. 2013). The cingulum bundle showed up prominently in a number of components in both hemispheres. Dorsally, components 28 and 49 in the left and 46 and 8 in the right hemisphere ran longitudinally between the corpus callosum and the cingulate sulcus, curving around the genu of the corpus callosum into perigenual cingulate cortex (Fig. 4a). The grey matter projections of these components also showed some loading on the temporal locations of the cingulum bundle, but the strongest loading of these parts of the cortex was on components 5 and 25 (Fig. 4b). Both of these ran along the medial part of the temporal lobe. Interestingly, their connectivity also weakly showed another limbic tract, the uncinate fascicle connecting anterior temporal cortex with parts of ventral prefrontal cortex. Posteriorly, the cortical hard parcellation showed two components in each hemisphere, one centered around the retrosplenial cortex and one more dorsally, that showed both connectivity to the cingulum bundle and to the thalamic radiations.

Parallel to the temporal part of the cingulum bundle we observed the fimbria of the fornix. The fornix was visible in symmetric components (c2 and c26; Fig. 4c) that emerged from the hippocampal area, curved upwards towards the splenium of the corpus callosum, and then ran close together to form the body of the fornix. At a lower threshold, these two components encompassed most of the thalamus around which the fornix courses. The fornix and cingulum were clearly dissociable in the temporal cortex, with the cingulum running ventrally to the fornix (Fig. 4d).

### Temporal and occipital components

**Fig. 5:**
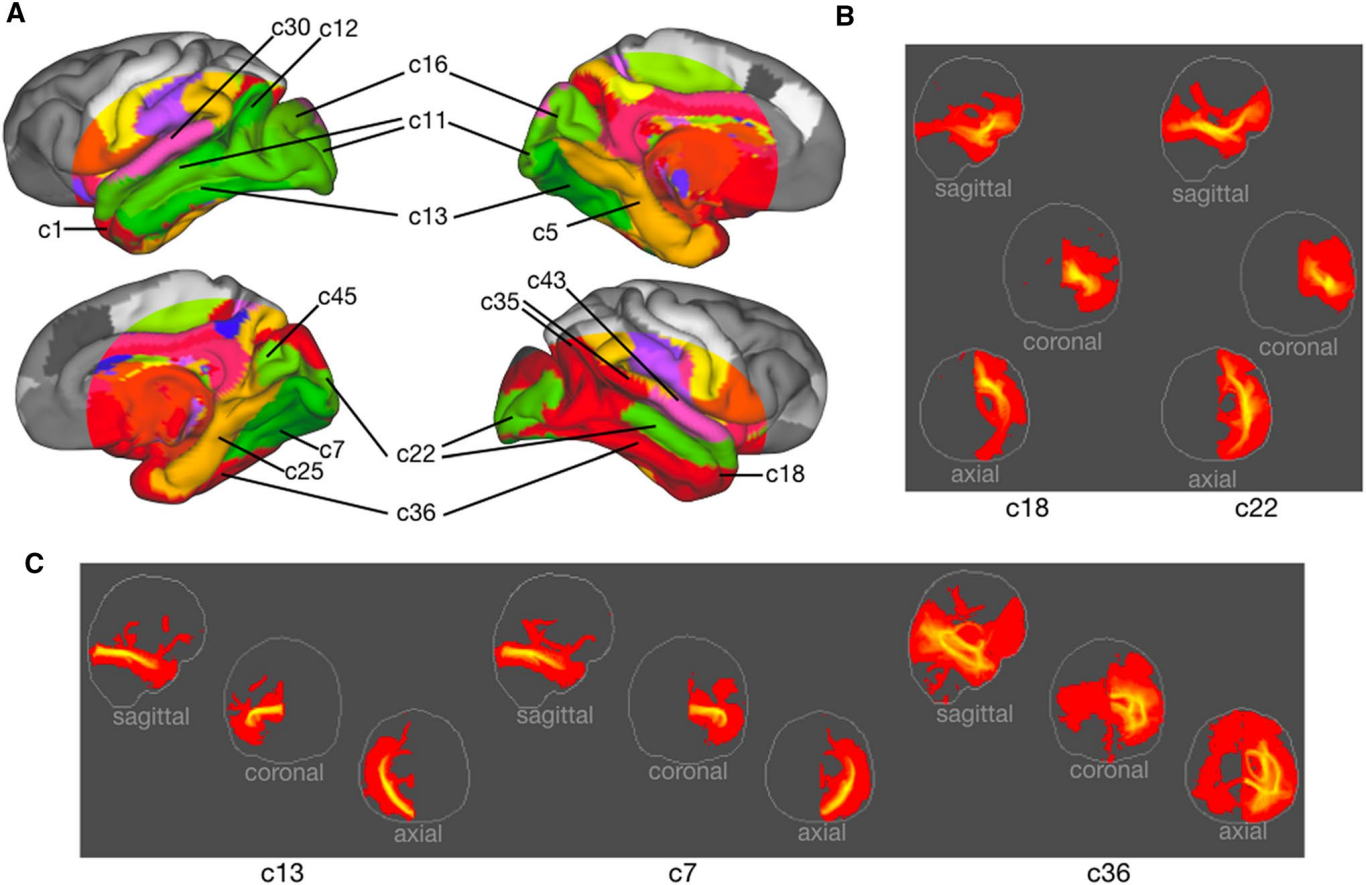
Temporal and occipital components. **a** Cortical hard parcellation as in Fig. 2 tilted and annotated to show temporal and occipital components in both hemispheres. **b** Maximum intensity projections for components showing connectivity patterns mostly reminiscent of the uncinate fascicle (c18) and the inferior fronto-occipital fascicle (c22). **c** Maximum intensity projections for components showing connectivity patterns mostly reminiscent of the inferior longitudinal fascicle

The superior part of the temporal lobe preferentially loaded on two components, both weakly symmetric, on the middle and superior part of the superior temporal gyrus. This cortical territory is known to show distinct organization between the human and macaque brain, due to cortical expansion and changes in the projections of the arcuate fascicle (AF) (Rilling et al. 2008; Van Essen and Dierker 2007), which means that assignment of white matter in this part of the chimpanzee cortex should be considered with caution. The anterior c30 in the left and c43 in the right hemisphere were most similar to the body of the middle longitudinal fascicle (MdLF) in other primates, covering part of Bailey’s area TA, while the posterior c12 and c35 had aspects of both MdLF and AF in its proximity to the auditory core (Fig. 5a).

The anterior part of the temporal lobe was reached in both hemispheres by components that showed highest weighting in a large part of the ventral medial prefrontal cortex, including Bailey’s areas FE, FG, and FH (c1, c18; Fig. 5a). The shape of their volume projection suggests that these components captured the limbic connections between the amygdala and anterior temporal cortex and the ventromedial frontal cortex that in the human and macaque are carried by the medial amygdalafugal pathway (AmF) and the uncinate fascicle (UF) (Folloni et al. 2017). At a lower threshold components also loaded on what looks like the inferior fronto-occipital fascicle (IFO) (Fig. 5b).

A profile much more reminiscent of the IFO was found in c22 in the left hemisphere and to a lesser extent c11 in the right and c43 in the left hemisphere. In humans, the IFO runs between orbitofrontal and frontopolar cortex and ventromedial occipital cortex (Forkel et al. 2014) and such a pattern is evident in these components as well. Noticeable as well, however, is that these components also showed the highest loading for the middle temporal gyrus. In both hemispheres, components more reminiscent of the IFO tended to reach more dorsal prefrontal territories than components more reminiscent of the UF. We also observed two highly symmetric components that are reminiscent of an insular branch of the IFO (cf. Maldonado et al. 2013).

The ventral surface of the temporal and occipital lobes shows a strongest weighting on c7 in the right hemisphere and c13 in the left. Both components show a course that is reminiscent of the inferior longitudinal fascicle (Fig. 5c). The ventral part of the temporal cortex of these components hits parts of the chimpanzee equivalent of the human fusiform gyrus (Bryant and Preuss 2018). In both hemispheres the component ran along the inferior temporal gyrus, but in the left hemisphere the anterior section of this part of the brain loaded highest on c36. This component showed an extensive mixture of various tracts, including some reminiscent of ILF and the occipital radiation (Fig. 5c). Components 16 and 45 in the dorsal part of the occipital cortex showed connections with the splenium of the corpus callosum.

### Dorsal components

**Fig. 6:**
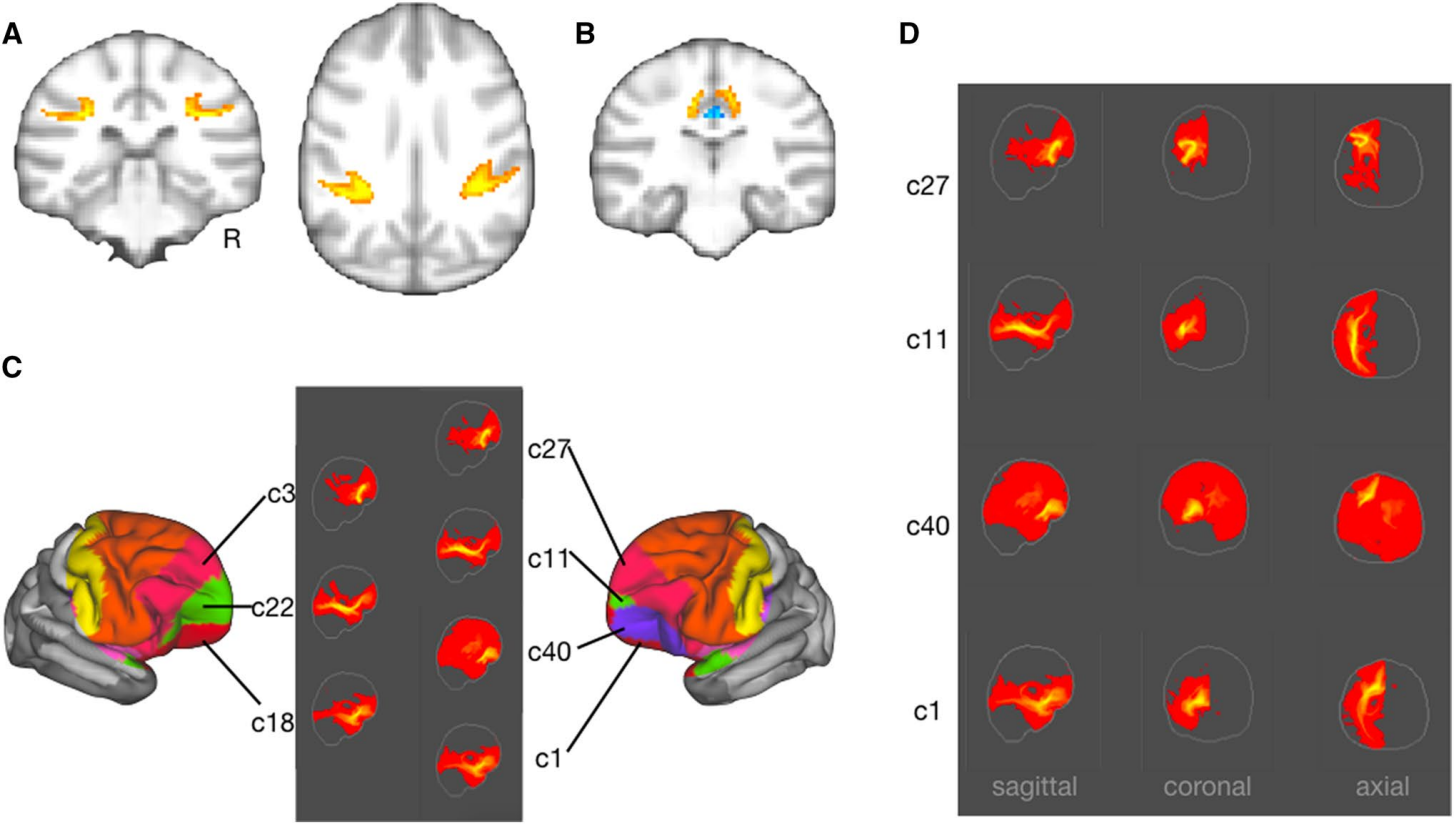
Dorsal components. Thresholded connectivity patterns of **a** components 15 and 41 around the fundus of the intraparietal sulcus and **b** components 17 and 33 (yellow) in comparison with dorsal cingulate components (blue). **c, d** Anterior frontal components show combination of UF and IFO-like connectivity in their maximum intensity projections

In contrast to the abundance of components weighting high on ventral longitudinal white matter, very few superior longitudinal tracts were visible. In macaques there is a complex of longitudinal fibres connecting frontal cortex with parietal and superior temporal cortex (Schmahmann and Pandya 2006). We did not observe many components encompassing parts of both parietal and frontal cortex, although some components did show white matter connectivity consistent with these tracts. For instance, behind somatosensory cortex we observed the symmetric components 15 and 41. Their highest loading is on white matter around the fundus of the intraparietal sulcus (Fig. 6a). Because of these characteristics we tentatively label them as part of the larger SLF complex. Slightly more posteriorly, territory both inferior and superior to the intraparietal sulcus was assigned to single components (c21 and c23) that seemed to originate in the thalamus and that we thus tentatively label as part of the thalamic radiation.

**Fig. 7:**
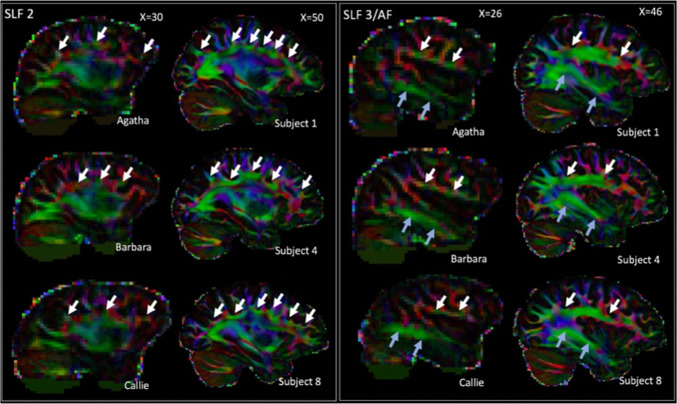
Illustration of SLF2 and SFL3/AF in the human and chimpanzee brain. Dorsal longitudinal pathways are generally more prominent in the human brain, as illustrated by the mostly continuous pathways in three representative human subjects (right columns compared to three representative chimpanzee subjects (left columns)

The lack of clear dorsal longitudinal fibres belonging to the superior longitudinal and arcuate fascicles could be considered surprising, since they have been reported by previous studies using tractography seeded in the dorsal white matter (Hecht et al. 2015; Rilling et al. 2008). It should be noted that the previous human study also reported only the second branch of the SLF and the AF, but not the first and third branches of the SLF. The AF/SLF run through a part of the brain where a number of different fibre bundles cross and it is conceivable that the current method seeding at the cortical surface has more difficulty reconstructing these fibres. To investigate whether the dorsal fibres in particular suffer more from these crossing fibres in the chimpanzee, we investigated the single subject principal diffusion direction maps in our data and that of single human subjects.^1^ Three representative subjects of both species are displayed in Fig. 7. This shows that the SLF2 and SLF3/AF are much clearly distinguishable in the human than in the chimpanzee. Thus, we conclude that the dorsal longitudinal fibres are less prominent in the chimpanzee, which accounts for our current lack of observation of these tracts.

Clearer evidence for dorsal longitudinal connectivity was provided by components 17 and 33 which showed strongest weighting on the dorsal medial surface. The core of the white matter associated with these components is just lateral to the dorsal part of the cingulum bundle (Fig. 6b). This could be interpreted as a dorsal extension of the cingulum or, based on its location in the macaque, the superior fronto-occipital fascicle (Schmahmann and Pandya 2006). Markis and colleagues identified a superior fronto-occipital fascicle in the human brain, but its location was more ventral than we observed here in the chimpanzee (Makris et al. 2007) and connectivity is with medial parietal cortex whereas the component’s main grey matter territory is mostly anterior of the callosalmarginal sulcus. Alternatively, this tract might be a part of the larger complex of superior longitudinal fasciculi.

The largest components with preferential weighting of lateral frontal cortex overlapped with Bailey’s area FB and part of FC (orange in Fig. 6c). These components’ connectivity seemed to consist of a conjunction of different tracts, rather than identifying distinct systems. This suggestion is supported by the fact that the two components showed only weak symmetry.

Anterior to this large patch, a series of components showed connectivity reminiscent of UF or IFO (Fig. 6c, d). Around the ventral part of the sulcus rectus we observed the extension of the IFO components described above as reaching occipital cortex and middle temporal gyrus. In contrast, components ventral to these IFO components (c1 and c40 in the left hemisphere, c18 in the right hemisphere) showed a connectivity profile more reminiscent of UF, reaching anterior temporal cortex but not extending as far posteriorly as IFO even in unthresholded images. Loading highest on the territory around the middle part of the inferior frontal sulcus and extending dorsally into the territory anterior to the middle fontal sulcus, we observed the symmetric components 27 and 3. The connectivity profiles showed a focus on more ventral cortical territory, each showing a distinctive shape, from the frontal operculum curling around to reach a termination in the territory anterior to the fronto-orbital sulcus.

## Discussion

We here present to our knowledge first assessment of the grey and white matter organization of the entire cerebral cortex of the chimpanzee. We were able to identify many of the main limbic, projection, and association fibre systems that are known from the macaque and human primates. Overall, the organization of the white matter shows much similarity with that of the human, with the organization of the temporal and temporal–frontal systems in particular providing clues on the specializations that have occurred in the shared lineage of great apes and humans.

As one of our closest living relatives, the chimpanzee is of obvious interest for understanding human cortical evolution. While detailed modern maps are available of the organization of the macaque and marmoset monkeys, both commonly used as model species in research, cortical maps of the chimpanzee generally date from before the era of contemporary neuroscience (Bailey et al. 1950; Campbell 1905; Walker 1938). Modern neuroimaging techniques allow renewed investigations into the great ape brain, especially with novel methods that permit imaging of postmortem tissue (see Mars et al. (2014) for a review). These techniques are providing much needed new data to debates on the human specialization in connectivity (Mars et al. 2018c; Rilling et al. 2012), cortical expansion (Donahue et al. 2018), and comparison between different model species (Schaeffer et al. 2017). In the great ape, pioneering studies have investigated specific systems (Bryant et al. 2018; Hecht et al. 2015; Rilling et al. 2008), but to date have not presented a comprehensive analysis of the whole cortex. The combination of publicly available data and novel analysis techniques allowed us to perform this exploratory study. Our results will inform future hypothesis-driven atlases and provide input for like-for-like comparisons with brain organization of other species.

The cingulum bundle was prominent in a number of components. It was noticeable that the dorsal and temporal parts of the bundle were for the most part visible in different components. This follows the observation made by Heilbronner and Haber (2014) in macaques, that brain regions often send connections only through part of the cingulum bundle. Some recent tractography studies have subsequently chosen to delineate distinct dorsal and ventral parts of the cingulum bundle when reconstructing this tract in the human and the macaque, e.g., Mars et al. (2018c), and it seems prudent to adopt a similar strategy in the chimpanzee. In humans, the cingulum bundle and the fornix follow distinct paths along the temporal lobe, connecting, respectively, to the amygdala and the hippocampus (Catani et al. 2013). A similar dissociation was observed here, with the two bundles belonging to distinct components.

The connectivity of components loading strongest on vertices of the temporal and occipital lobe was reminiscent of several longitudinal tracts identified in other primates. We found evidence of a dorsal tract in the superior temporal gyrus similar to the middle longitudinal fascicle (Makris et al. 2009), a fronto-temporal tract similar to the inferior fronto-occipital fascicle (Forkel et al. 2014; Mars et al. 2016a), and a tract similar to the inferior longitudinal fascicle. The latter was evident in components that loaded highly on the ventral surface of the temporo-occipital cortex in an area possibly homologous to human fusiform gyrus (Bryant and Preuss 2018). The human ILF is thought to have a branch that serves this part of the cortex (Latini et al. 2017).

As is the case for the human, the chimpanzee temporal cortex has a superior, inferior, and middle temporal gyrus, whereas the macaque only has two distinct lateral gyri on either side of the superior temporal sulcus. The homology of the different parts of human, chimpanzee, and macaque temporal cortex remains a partly open question, but it is known that temporal cortex has differentially expanded and reorganized since the last common ancestor of humans and macaques (Mars et al. 2013; Rilling and Seligman 2002). We observed MdLF and ILF-like components in the superior and inferior temporal gyri of the chimpanzee, but the middle temporal gyrus showed the most strongest loading on components with connectivity profiles most similar to the IFOF or to a mixture of IFO and MdLF. The IFO is very prominent fibre bundle in the human brain, but its strength in the macaque monkey is more controversial (Mars et al. 2016a; Takemura et al. (2017); but see Decramer et al. 2018). Overall, the chimpanzee temporal lobe appeared similar in organization to that of the human, with the notable absence of a strong arcuate fascicle reaching the middle and inferior temporal cortex. From previous work in the humans, it is known that the exploratory technique employed here is sensitive enough to pick up the arcuate fascicle in humans (O’Muircheartaigh and Jbabdi 2018). Our results are thus consistent with prior evidence that the temporal lobe extension of the arcuate fascicle is a human specialization (Rilling et al. 2008). A further explicit comparison of the tract architecture of temporal lobe regions might shed light on the homology of different parts of the temporal cortex across monkeys, apes, and humans.

It was noticeable that we did not observe strong parietal–frontal components, even though it is known that these systems are interconnected through a series of parallel pathways (Caminiti et al. 2017; Vijayakumar et al. 2018). A series of longitudinal white matter pathways are thought underlie many of these connections, with most authors distinguishing three branches of the superior longitudinal fascicle (SLF) as well as the arcuate fascicle (AF) (e.g., Makris et al. 2005; Schmahmann and Pandya 2006; Thiebaut De Schotten et al. 2011). Although we found some components that are reminiscent of some of these tracts, clear parietal–frontal components were not detected. Indeed, we observed that this part of the brain had the lowest consistency across hemispheres, suggesting that the parcellation was least reliable. This could partly be due to the tractography method. Parietal–frontal connections are generally hard to resolve due to the large amount of crossing fibres from the longitudinal tracts, the corona radiata, and the corpus callosum (Behrens et al. 2007). The relatively low b-value of the dataset might have contributed to the difficulty in resolving these different fibre directions. Consistent with this observation, our 50 independent components explained 22% of variance, which is more comparable to human developmental data than to the human adult data (O’Muircheartaigh and Jbabdi 2018). However, it has also been observed that dorsal longitudinal tracts in general are much weaker—and therefore more difficult to reconstruct—in the macaque than in the human and that this might have functional consequences for language (Eichert et al. 2018; Rilling et al. 2008) and social learning behavior (Hecht et al. 2013). We might be picking up the consequences of a similar effect in the chimpanzee.

The inferior frontal cortex of the great ape has a notably different shape and gyrification than the human brain (Connolly 1950). Nevertheless, we noticed that for a large part it is reached by tracts that are similar to those in the human. Connections from the temporal cortex similar to the IFO, connecting frontal cortex with middle temporal gyrus and occipital cortex, and the UF and AmF, reaching anterior temporal cortex and amygdala, were clearly distinguishable. Interestingly, the connectivity of the components containing UF seemed to extend to more dorsal territory than is the case for macaque, similar to what has been observed for the human (Folloni et al. 2017; Thiebaut de Schotten et al. 2012). Although human frontal lobes have unique specializations, including morphometric changes (Bruner and Holloway 2010), increased gyrification, and greater white matter volume (Rilling 2006), there is also evidence for important similarities between human and chimpanzees. For instance, chimpanzee frontal cortices display some expansion of area 10 (Semendeferi et al. 2001), overall expansion (Semendeferi et al. 2002), and increased white matter (Smaers et al. 2011) compared to monkeys. Connectivity profiles of different parts of the cortex are a promising avenue to match cortical areas across species by abstracting away from features such as brain size and shape (Mars et al. 2018a) and the evidence presented here suggests that, although of a different shape, the chimpanzee frontal cortex shows many organizational principles similar to that of the human.

Future work will focus on explicit comparisons between the whole-brain organization of the chimpanzee brain with that of the macaque and the human. We have recently proposed a method to do this based on homologous white matter tracts (Mars et al. 2018c). By defining the bodies of the tracts in each species and expressing each cortical vertex in terms of the connectivity fingerprint of all white matter tracts, one can in effect create a common connectivity space for all three brains (Mars et al. 2018b). However, for this approach to work one needs to have some understanding of the white matter architecture of each brain. The current, explorative study provides a first step in this direction.

We have chosen here to perform our tractography from the cortical surface towards the brain’s white matter. This approach allowed us to visualize our results in terms of grey matter networks driven by underlying white matter connectivity. It might also be argued that this approach is less prone to the gyral bias sometimes seen in tractography (Schilling et al. 2017) compared to the alternative approach of tracking towards the cortical surface. However, our results should be interpreted in the light of this strategy. It means that the white matter networks we identified do not necessarily correspond directly to single white matter tracts as would be identified using traditional tracer studies (e.g., Schmahmann and Pandya 2006) or tractography approaches aimed specifically at reconstructing particular fibre pathways (e.g., Makris et al. 2005; Thiebaut de Schotten et al. 2012). In some cases, white matter tracts were represented in more than one component; for example, two components along the dorsal part of the cingulum bundle, presumably due to seeding on the different parts of the cingulate gyrus, and a third representing the temporal extension. In other cases, two adjacent fasciculi were captured in a single component, as in the case for the reconstruction of the IFO and MdLF. In several cases, we observed nearly one-to-one mapping of components and recognized fibre bundles (ILF, corticospinal tract, fornix). Finally, some tracts were not wholly represented by the components; a posterior segment of the SLF complex appears to have been reconstructed, but without coherent connections to frontal regions.

## Conclusion

Although our approach does not reproduce all major white matter bundles in the same form as canonical fascicular maps, it does permit us to form a first impression of the organization of the chimpanzee cerebral cortex. Comparatively little is known about cortico-cortical connectivity or fascicular organization in chimpanzees, and as a result determining structural homologies with humans and macaques is an ongoing challenge. By harnessing a data-driven approach to understanding white matter organization, we avoid making a priori assumptions about these structures. Here, we endeavour to provide a first global assessment of grey matter networks and their associated connectivity patterns of chimpanzee cortex. This method can be implemented on other species and directly compared with data from humans to assist in determining inter-species homologies and, in turn, offer greater insight into the evolution of human and hominoid brains.

## Footnotes

1 Human in vivo diffusion MRI data were provided by the Human Connectome Project (HCP), WU-Minn Consortium (Principal Investigators: David Van Essen and Kamil Ugurbil; 1U54MH091657) funded by the 16 NIH Institutes and Centers that support the NIH Blueprint for Neuroscience Research; and by the McDonnell Center for Systems Neuroscience at Washington University (Van Essen et al. 2013). Minimally preprocessed datasets from the Q2 public data release were used. Data acquisition and preprocessing methods are detailed in Uğurbil et al. (2013), Sotiropoulos et al. (2013), and Glasser et al. (2013).
